# Punishment to Support: The Need to Align Animal Control Enforcement with the Human Social Justice Movement

**DOI:** 10.3390/ani10101902

**Published:** 2020-10-16

**Authors:** Sloane M. Hawes, Tess Hupe, Kevin N. Morris

**Affiliations:** Institute for Human-Animal Connection, Graduate School of Social Work, University of Denver, Denver, CO 80208, USA; sloane.hawes@du.edu (S.M.H.); tess.hupe@du.edu (T.H.)

**Keywords:** animal control, policy, one health, one welfare, humane communities, social justice, access to care, underserved communities, companion animals

## Abstract

**Simple Summary:**

The current emphasis on enforcement and punishment in animal control policy has disproportionately negative impacts on low-income communities in the United States (US), particularly people of color. In this way, animal protection efforts are perpetuating many of the same inequities under examination in the human social justice movement. Reallocating the resources that have historically gone towards enforcement in communities to efforts that provide support in addressing the root causes of animal welfare concerns is needed to improve outcomes for pets in historically underserved communities.

**Abstract:**

Due to inherent and systemic biases, animal control policies in the US are over-enforced in low-income communities and communities of color, resulting in worse health outcomes for the pets in these communities. These outcomes are exemplified by higher confiscation, relinquishment, and euthanasia rates, lower return to owner rates, and extended lengths of stay in animal shelters. The Humane Communities framework operationalizes One Health and One Welfare concepts to comprehensively address issues of inequity at both the individual and structural levels to improve animal control policy and outcomes. Person-centered and culturally competent policies and programs that focus resources on addressing root causes of pet health and welfare issues as opposed to an emphasis on code enforcement can create more positive, scalable, and sustainable improvements in human, other animal, and environmental health and welfare outcomes. This shift from punishment-oriented approaches to support-based models of animal control aligns the animal welfare field with the modern human social justice movement.

## 1. Introduction

The role of animal control agencies in the United States (US) has evolved over the last century. Modern animal control and humane law enforcement officers engage in policing of communities to “correct community problems resulting from irresponsible animal ownership” [1]. Examples of the ordinances enforced by animal control agencies include: licensing and rabies vaccination requirements; leash laws; anti-tethering laws; monitoring of “at-risk” and “dangerous” animals (typically dogs); licensing of feral cat colonies; regulations for adequate care of animals; regulations for the number of animals an individual can keep (to prevent potential “hoarding” of animals); and investigation of cruelty, abuse, and neglect cases [2,3]. Previous studies have refuted the assertion that animal cruelty laws are uniquely exempt from the racism that is pervasive throughout other areas of US criminal law [4]. By defining the standards for “responsible pet ownership” in vague language (e.g., “necessary” or “sufficient”), that is highly subjective and largely unobtainable for anyone in the US other than white, middle and upper-class individuals, animal protection ordinances, similar to human criminal justice policies, disproportionately target communities of color with their enforcement interventions [5,6,7,8]. Therefore, animal control agencies should examine if the enforcement of their policies is laden with implicit bias and if their policies reflect a recognition of how the circumstances surrounding an animal welfare issue (providing shelter, behavioral training, or veterinary care) might be driven by the very same structural inequities and social determinants of health that influence health outcomes for socially disadvantaged human populations [4,9].

Animal control over-policing of low-income communities and people of color is a concern for two primary reasons. First, it means that modern animal control policies are perpetuating many of the same issues under examination in the human social justice movement. Implicit bias and a failure to acknowledge the social determinants of health in the human criminal justice system has resulted in people of color representing a majority of drug-related arrests, people experiencing homelessness, and students receiving suspensions and expulsions in schools in the US [10,11,12]. Aligning animal services with the human social justice movement would require increased recognition of how pursuing animal welfare by advocating for punitive outcomes, such as incarceration, deportation, confiscation, and criminal registration, perpetuates a system that disproportionately targets low-income communities and people of color [4,5]. Instead, the human social justice movement advocates for identifying programs and policies that enhance the resilience of a community by addressing the root causes of human welfare concerns (e.g., addressing discriminatory attitudes, increasing access to healthcare, providing high-quality education). Second, these policies create and perpetuate worse health outcomes for the pets that animal control policies are designed to protect. Confiscating animals from their homes and impounding them in animal shelters negatively impacts animal control agencies’ capacity to serve animals in crisis and contributes to higher euthanasia rates, lower return to owner rates, and extends lengths of stay in animal shelters [6,13]. Broader approaches that build upon the capabilities of individuals and communities to comprehensively address human, other animal, and environmental issues are needed to improve community health and welfare effectively and sustainably.

## 2. One Health and One Welfare in Animal Control Reform

The human–animal bond is largely believed to improve the health and welfare of people and pets [14,15,16]. However, there are a number of interrelated factors that inform poor human and pet health outcomes in communities, including individual factors (e.g., household income, race/ethnicity, and mental health) and structural factors (e.g., over-policing, lack of access to healthcare, and housing discrimination) [17,18,19]. A previous study examining best practices in animal control found that most cities’ animal control ordinances emphasize code enforcement over actual human or animal health and welfare outcomes [3]. This finding identifies a major gap in the ability of animal control policies to account for broader factors (e.g., socioeconomic status, educational attainment, and built environment) that influence animal welfare in a community. Addressing this gap in animal control policy would result in a set of policies that can effectively and sustainably address animal welfare issues. The One Health and One Welfare frameworks were developed to “address current and potential health and welfare issues” through interdisciplinary collaboration and recognize that the health and welfare of humans, other animals, and the natural environment are interconnected [20,21]. These frameworks can inform animal control policy reform in that they acknowledge the influence human wellbeing and environmental conditions have on the community’s animal health and welfare outcomes.

The Humane Communities framework was developed to highlight how integrating expertise across multiple disciplines to identify best practices and to assess the impacts of policies or programs on the health and wellness of humans, other animals, and the environment can be used to operationalize the One Health and One Welfare concepts [22]. While an initial series of social-economic impact studies of animal welfare policies and practices have been used to explore how the Humane Communities framework could guide policy development, more research is needed to assess specific practices that can most effectively and sustainably improve outcomes across the One Health and One Welfare triads [8,23,24].

There are substantial barriers to implementing animal control policies that promote One Health and One Welfare, including inherent biases regarding how and why individuals living in poverty may require additional support resources (e.g., they are just “lazy” and need to get a job so they can pay for their pet’s care on their own, rather than relying on government handouts); the animal welfare field’s historic commitment to a specific definition of “responsible pet ownership” that is driven by racism, classism, and the White dominant culture; an absence of strategies for engaging with marginalized populations in a culturally competent manner; over-policing in communities of color; lack of transparency and oversight in data regarding enforcement; lack of a concerted effort to address structural barriers to accessing pet support services; lack of animal control officer training to perform basic animal handling and zoonoses prevention tasks or in de-escalation strategies; and limited funding opportunities for projects aimed at achieving One Health and One Welfare outcomes [4,5,25,26,27,28,29,30]. Without identifying specific strategies for overcoming each of these barriers, the implicit bias that is present in animal control policy will continue, resulting in disproportionately negative impacts on the pet owners of color and their pets that live in low-income communities.

## 3. Humane Communities and the Shift from Punishment to Support

The Humane Communities framework addresses animal welfare policymaking and program implementation at two levels. First, it advocates for integrating One Health and One Welfare considerations into program evaluation and policy-making discussions [30]. For example, metrics, such as “Live Release Rate”, are currently used by animal welfare organizations to inform program development and to hold organizations accountable for the number of animals that die or are euthanized while in the custody of animal shelter or rescue organizations. However, these metrics lack One Health and One Welfare considerations, including the harm done to pets, pet owners, and their families when animals are removed from their homes [30,31]. Alternative metrics for evaluating animal welfare programs that capture a community’s capability to promote the interconnectedness of human, other animal, and environmental outcomes (e.g., referrals to support services, improvements in the human–animal bond, and increasing environmental stewardship attitudes) are needed. Second, the Humane Communities framework emphasizes person-centered approaches to community-based work that focus on culturally competent strategies that recognize the complexity of factors informing human, other animal, and environmental health and welfare issues. An example of this approach relevant to the discussion of animal control policy reform is the replacement of punishment-oriented methods with support-based models.

Punitive approaches to addressing social problems, such as citation and incarceration, often blame the individual while disregarding the structural causes of an issue. For example, an officer might encounter a dog in the field that appears to have a case of mange and is also covered in motor oil. In one version of this scenario, the animal control officer assumes that the owner is neglecting their pet dog by allowing the mange to develop (i.e., “wanton” lack of veterinary care) while also committing cruelty by covering the dog in motor oil. Under the law, the officer then issues a cruelty citation to that individual for “failure to provide veterinary care.” But if that officer were to consider the structural causes behind the dog’s condition, such as a lack of low-cost options for veterinary care, lack of necessary supplies (e.g., leash or carrier) and transportation to get to the vet, or lack of information regarding best practices for treating mange, then that officer might recognize that the owner had actually attempted to treat their dog using a common home remedy for mange and not be so inclined to jump to a punitive approach for addressing this animal welfare concern. This approach could apply to other circumstances that are routinely charged as animal cruelty/neglect (e.g., emaciation that could be resolved with dewormer or lack of shelter that could be resolved by providing a new doghouse). It is important to note that this supportive approach does not replace enforcement for actual cruelty and neglect. Rather, it encourages first considering solutions that might address the root cause of the animal welfare issue while keeping the animal in their home, before considering punitive measures, such as citations or confiscation. These examples illustrate how the interpretation of the language contained in the ordinance allows enforcement of animal control policy to be subject to an individual officer’s implicit bias. Due to structural inequities that inform whether an individual will have access to pet support services, this lack of distinction in animal control policy between actual cruelty and neglect cases and issues driven by barriers to accessing information or pet support services has resulted in a systemic bias towards individuals who have limited access to such services (e.g., low-cost veterinary care, behavior support, pet-friendly housing).

In contrast, human social services address human welfare concerns by providing critical safety net programs, such as social security, housing vouchers, and food assistance, that support the basic needs of vulnerable individuals and their families so they can focus on longer-term strategies for maintaining their wellbeing. Research from several different human social support systems, including education, substance use treatment, and housing security, has demonstrated how models that focus on addressing root causes are more effective at creating positive and sustained community health outcomes than punitive approaches, particularly when the most vulnerable individuals and families in a community are prioritized to receive the support they need [32,33,34,35]. For example, some schools have shifted away from punitive zero-tolerance discipline policies and adopted supportive approaches, such as restorative justice. There is preliminary evidence suggesting that these approaches contribute to a more positive school climate, improvements in student relationships, community engagement, academic achievement, staff support, and decreases in fighting, bullying, violence, suspensions, and expulsions [32,33]. Supportive interventions for substance use have also been developed as alternatives to the criminal justice system, including harm reduction approaches (e.g., increasing naloxone availability, promoting needle exchange, expanding medication-assisted addiction treatment, increasing psychosocial treatment, and outreach programs), which have been found to improve recovery from addiction, increase lifespan, enhance the quality of life, and decrease the number of deaths [34]. This focused and proactive safety net approach to animal service is largely absent in the field of animal control and humane law enforcement.

An example of an animal welfare program that is operationalizing the shift from punishment to support is the Humane Society of the United States Pets for Life program, which is working to redefine the role of animal services as one component of a robust system of support for vulnerable communities. Pets for Life is a national program that has operated in more than 40 communities across the US since 2011 and advocates for people who are routinely overlooked or looked down upon in the animal welfare field [36]. At the individual level, Pets for Life proactively engages people within their community through proactive direct care that provides ongoing and comprehensive owner support services (e.g., spay/neuter, flea/tick preventive, dewormer, microchipping, rabies, parvo, and distemper vaccinations, minor surgeries, antibiotics, grooming, skin treatment, collars/leashes, outdoor pet shelters, food/treats, and litter box), rather than punishing individuals for lack of access to services or waiting until individuals and pets are in crisis to provide support [36]. At the broader community level, the Pets for Life program addresses the root causes of animal welfare concerns through mentorship and training for community-based animal service providers and advocating for policy reform [36]. There are several other programs operationalizing examples of the shift from punishment to support, including the University of Tennessee–-Knoxville Program for Pet Health Equity’s Align Care program, which was developed to address the issue of access to affordable veterinary care, and American Pets Alive’s Human Animal Support Services program, which was developed to “reimagine what animal protection is in terms of protecting the public and animals in the community” [37,38].

Each of these programs, which are working to increase health equity for pets and their owners, have identified how direct care that proactively increases access to pet care supplies, veterinary services, and behavior support can promote positive health and welfare outcomes for pets and their owners. However, they will be limited insofar as the broader animal control policies of the community continue to criminalize individuals experiencing poverty and people of color who have pets. To address this issue, Pets for Life works with communities to develop animal control policy that aligns with the goal of improving outcomes across the One Health and One Welfare triads. These policy reforms include increasing return to field programs; eliminating return to owner fees; removing adoption requirements; offering owner present euthanasia; examining implicit bias in ordinance language, such as “intent” or “morality of the public”; decreasing the number of civil penalties; minimizing felony penalties for most animal-related offenses by individual pet owners; and training officers and outreach staff in effective and strengths-based community engagement [36]. In practice, these policies are exemplified by providing low-cost veterinary care or a doghouse rather than issuing a citation for “wanton malicious intent with failure to provide veterinary care” or “lack of shelter.” Ideal policies can be adapted based on the community’s specific needs and are responsive to the reality that many of the issues associated with acquiring pets (e.g., commercial breeders), free-roaming animals (e.g., feral or unowned dogs and cats), end of life care (e.g., access to owner present euthanasia services), and engagement with animal control agencies are driven by access to information or access to care issues, thereby positioning animal control agencies as resources for the community rather than adversaries.

The shift from punishment to support requires a reallocation of financial and staff resources that reflects a commitment to equity and justice and an investment in strategies that can achieve scalable and sustainable change. While this shift may be daunting for some agencies to consider, previous studies of human criminal justice policy reform efforts have indicated that communities in the US generally support policies that are supportive over punitive [39]. This approach can also be more cost-effective than a punitive approach. For example, Rochester Animal Services (Rochester, NY, USA) spends an average of $160 per animal served through Pets for Life, compared to an average cost of $300 per cat and $375 per dog if that animal were to be taken into the custody of the shelter [40]. Salt Lake County Animal Services (Salt Lake City, UT, USA) spends an average of $400 per animal to implement an enforcement approach that includes officer response, veterinary needs, in-shelter care, overhead, supplies, and pet placement. In contrast, the average cost per pet served through the Pets for Life model in Salt Lake County is $116 [36]. This shift from punishment to support by investing in services that keep pets in their current homes, instead of confiscating, sheltering, and rehoming them, is a scalable, sustainable, and generalizable model that animal control agencies across the US can implement for preserving the health of humans, other animals, and the environment in a community, regardless of community demographics, finances, or leadership.

## 4. Conclusions

The human social justice movement is working to increase recognition that our society cannot successfully address concerns over public health and safety without simultaneously addressing issues of social justice that limit the capabilities of communities [4]. Policy reform that is informed by the One Health and One Welfare frameworks can provide a more comprehensive approach to maintaining public health and safety that prevents further harm against underserved and socially disadvantaged populations, particularly low-income communities and people of color. Shifting animal control policies from punishment to support is intended to act on the recognition of the physical and emotional benefits of the human–animal bond and incorporating animal control agencies into a more robust system that supports pet ownership [36].
